# TB as a cause of hospitalization and in-hospital mortality among people living with HIV worldwide: a systematic review and meta-analysis

**DOI:** 10.7448/IAS.19.1.20714

**Published:** 2016-01-12

**Authors:** Nathan Ford, Alberto Matteelli, Zara Shubber, Sabine Hermans, Graeme Meintjes, Beatriz Grinsztejn, Greer Waldrop, Katharina Kranzer, Meg Doherty, Haileyesus Getahun

**Affiliations:** 1 Department of HIV, World Health Organization, Geneva, Switzerland; 2 Global TB Programme, World Health Organization, Geneva, Switzerland; 3 Clinic of Infectious and Tropical Diseases, WHO Collaborating Centre for TB/HIV and TB Elimination, University of Brescia, Brescia, Italy; 4 Department of Infectious Disease Epidemiology, Imperial College London, London, UK; 5 Desmond Tutu HIV Centre, University of Cape Town, Cape Town, South Africa; 6 Amsterdam Institute for Global Health and Development, Amsterdam, the Netherlands; 7 Clinical Infectious Diseases Research Initiative, Institute of Infectious Disease and Molecular Medicine, University of Cape Town, Cape Town, South Africa; 8 Instituto Nacional de Infectologia Evandro Chagas, Fundação Oswaldo Cruz, Ministry of Health, Rio de Janeiro, Brazil; 9 University of Maryland school of Medicine, Baltimore, MD, USA; 10 Department of Clinical Research, Faculty of Infectious and Tropical Diseases, London School of Hygiene and Tropical Medicine, London, UK; 11 National and Supranational Tuberculosis Reference Laboratory, Research Centre Borstel, Germany

**Keywords:** HIV/AIDS, hospitalization, morbidity, mortality, tuberculosis

## Abstract

**Introduction:**

Despite significant progress in improving access to antiretroviral therapy over the past decade, substantial numbers of people living with HIV (PLHIV) in all regions continue to experience severe illness and require hospitalization. We undertook a global review assessing the proportion of hospitalizations and in-hospital deaths because of tuberculosis (TB) in PLHIV.

**Methods:**

Seven databases were searched to identify studies reporting causes of hospitalizations among PLHIV from 1 January 2007 to 31 January 2015 irrespective of age, geographical region or language. The proportion of hospitalizations and in-hospital mortality attributable to TB was estimated using random effects meta-analysis.

**Results:**

From an initial screen of 9049 records, 66 studies were identified, providing data on 35,845 adults and 2792 children across 42 countries. Overall, 17.7% (95% CI 16.0 to 20.2%) of all adult hospitalizations were because of TB, making it the leading cause of hospitalization overall; the proportion of adult hospitalizations because of TB exceeded 10% in all regions except the European region. Of all paediatric hospitalizations, 10.8% (95% CI 7.6 to 13.9%) were because of TB. There was insufficient data among children for analysis by region. In-hospital mortality attributable to TB was 24.9% (95% CI 19.0 to 30.8%) among adults and 30.1% (95% CI 11.2 to 48.9%) among children.

**Discussion:**

TB remains a leading cause of hospitalization and in-hospital death among adults and children living with HIV worldwide.

## Introduction

Tuberculosis (TB) remains a leading cause of HIV-associated mortality and morbidity among both adults and children worldwide [1,2]. In 2013, an estimated 1.1 million of the 9.0 million people who developed TB worldwide were HIV-positive, and there were 360,000 deaths from HIV-associated TB [3]. Among people initiating antiretroviral therapy (ART), incident TB is associated with poorer virological outcomes and a higher risk of early mortality [4–6], with autopsy studies suggesting that TB is the cause of death in over a third (37%) of adult persons living with HIV (PLHIV) in sub-Saharan Africa [7].

Recent studies from both low- [8,9] and high-income settings [10,11] have reported that TB contributes substantially to hospitalization among people living with HIV, and mortality among those hospitalized is high [12]. The primary aim of this review was to determine the proportion of hospitalizations and in-hospital deaths among HIV-positive adults and children attributable to TB worldwide and by World Health Organization (WHO) geographic region.

## Methods

This study is a sub-analysis of data collected as part of a global systematic review of causes of hospitalization among HIV-positive patients. The full methodology of the study is explained elsewhere [12]. Briefly, two investigators (NF, ZS) working independently and in duplicate searched Medline via PubMed, Embase, Web of Science, LILACS, AIM, IMEMR and WPIMR from inception up to 31 January 2015. The investigators also screened all International AIDS Society conferences up to Melbourne, 2014. Consensus was achieved to resolve discrepancies. Investigators followed a study protocol available from the corresponding author; the search strategy is provided in the Supplementary file. Studies were considered eligible if they included TB among the reported causes of hospitalization within a cohort of at least 20 HIV-positive hospitalized patients. In order to focus on data following ART scale-up, we sought data reported after 1 January 2007; studies reporting causes of hospitalization prior to 2007 were included if the majority (>50%) of hospitalizations occurred after 2007. The same investigators extracted duplicate data on the number of HIV-positive patients hospitalized for TB and the number of patients who died of TB during hospitalization, among all HIV-positive hospitalized patients. Data were also extracted on study setting, patient age (adults and children, as defined by the studies), prior knowledge of HIV status, length of hospital stay, method of TB diagnosis and usage of ART and isoniazid preventive therapy (IPT) at admission. For all reported causes of hospital admission and in-hospital mortality, proportions and 95% confidence intervals were calculated. Data were pooled following transformation to stabilize the variance in the raw proportions [13,14] using random effects meta-analysis [15]. The analyses were conducted disaggregated by age (adults vs children) and WHO geographical region (www.who.int/about/regions/en/). Meta-regression was used to explore the potential influence of ART status and calendar year on TB hospitalization. For adult studies, subgroup analyses explored the potential influence of diagnostic method (there was insufficient information for this to be assessed for children). All data were analyzed in Stata version 12.0.

## Results

**Figure 1:**
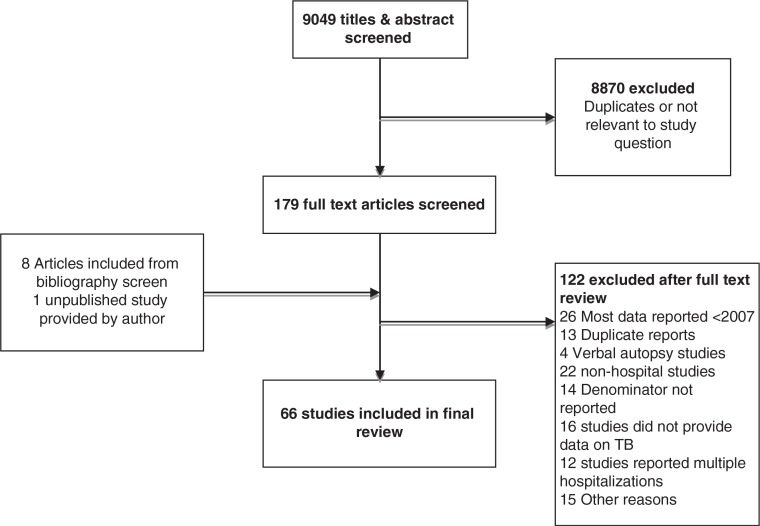
Study selection process.

From an initial screen of 9049 titles and abstracts, 66 studies were included in the final review. These studies were carried out in 42 countries, across all WHO regions (Figure 1). Characteristics of studies included in this review are summarized in the Supplementary file.

For adults, 51 studies reported 5497 cases of TB as a cause of hospitalization among 35,845 adult patients. Studies were carried out in 42 countries across all regions. Among all HIV-positive hospitalized patients, median age was 37 years (interquartile range [IQR] 35 to 40 years), median length of stay was 12 days (IQR, 7 to 16 days) and median CD4 at hospitalization was 131 cells/mm^3^ (IQR 76 to 199 cells/mm^3^). The proportion of adults on ART at time of hospitalization did not change over time. Pulmonary TB was mainly diagnosed by clinical assessment supported by at least one laboratory finding (65% of studies reporting this information), whereas for children diagnosis was generally based on clinical diagnosis alone (Supplementary file). Clinical diagnosis of extrapulmonary TB in adults was variously supported by examination of cerebrospinal fluid, non-response to presumptive broad-spectrum antibiotic treatment, radiological examination and lymph node biopsy. Around a third (31.9%) of all adult patients were newly diagnosed as HIV-positive at the time of hospitalization. Only two studies reported use of IPT; both reported that no patients were on IPT at time of admission.

**Figure 2:**
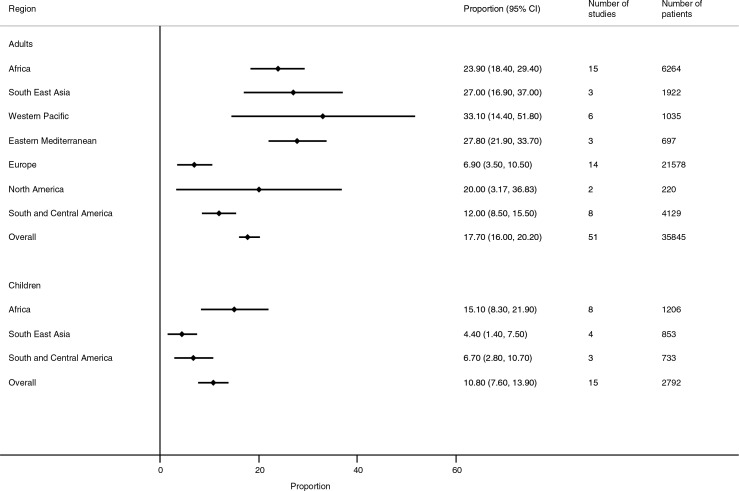
Proportion of hospitalizations among HIV-positive patients that were because of tuberculosis.

The overall proportion of adult hospitalizations because of TB was 17.7% (95% CI 16.0 to 20.2%) with regional variations (Figure 2). This proportion was 22.3% (95% CI 15.8 to 28.8%) when the analysis was restricted to studies in which TB was diagnosed by clinical assessment supported by at least one laboratory finding. Two-thirds of these hospitalizations were because of pulmonary TB (66.6%; 95% CI 55.7 to 77.5%). A high level of within-region variability was also observed; for example, in the African region, TB as a cause of hospitalization ranged from 8.1% to 41.0%. In order to assess whether the overall result was influenced by the inclusion of older studies, we ran a sensitivity analysis in which all studies reporting data prior to 2009 were dropped from analysis; the proportion of patients hospitalized because of TB was not significantly different from the overall result (23.8%; 95% CI 18.0 to 29.6%).

Twenty seven studies reported cause of death. There were 2376 deaths reported among 16,647 hospitalized adults (pooled proportion for deaths among those hospitalized: 24.9%; 95% CI 19.0 to 30.8%), among which 620 were because of TB (pooled proportion of TB deaths among all deaths: 27.3%; 95% CI 21.4 to 33.3%); 29.3% (95% CI 20.2 to 38.4%) of adults hospitalized for TB were reported to have died of TB. These results did not differ when the analysis was restricted to studies in which TB was diagnosed by clinical assessment supported by at least one laboratory finding.

Twenty seven studies reported ART status among adults at admission. The median proportion on ART was 37.5% (IQR 13.8 to 45.5%). In meta-regression, the proportion of adult patients hospitalized for TB decreased as ART coverage among those hospitalized increased (*p*<0.001). There was no change in the proportion of hospitalizations because of TB over time.

For children, 15 studies reported 292 cases of TB as a cause of hospitalization among 2792 children across 15 countries, all in low- and middle-income settings. Median length of stay was 10 days (IQR 9 to 11 days). Median age was five years (IQR 3 to 7 years). Only a minority of studies (5/14) reported CD4 cell count: the median ranged from 151 to 845 cells/mm^3^. Over a quarter (26.9%) of all paediatric patients were newly diagnosed as HIV-positive at the time of hospitalization. As for adults, the proportion of children on ART at time of hospitalization did not change over time.

The proportion of hospitalizations because of TB was 10.8% (95% CI 7.6 to 14.0). There was only one documented case of extrapulmonary TB. ART coverage among those hospitalized was associated with a lower proportion of TB as cause of admission, but this did not reach statistical significance because of the limited distribution of data (i.e. most children were on ART at admission). Overall, 431 hospitalized children died (pooled proportion for deaths among those hospitalized: 18.8%; 95% CI 8.1 to 29.5%); among whom 30.1% (95% CI 11.2 to 48.9%) died of TB; 45.4% (95% CI 6.0 to 84.2%) of children hospitalized for TB died of TB.

## Discussion

There has been a remarkable increase in access to ART for PLHIV over the past decade, with over 15 million people receiving treatment by mid-2015 [16]. HIV testing coverage has also increased, and people living with HIV are accessing treatment earlier in their disease progression [17]. Nonetheless, our review demonstrates that TB remains a leading cause of hospitalization among PLHIV, particularly in Africa, South East Asia, and the Western Pacific regions, highlighting the important continuing contribution of TB as a major cause of serious HIV-associated morbidity. This was consistent across all regions, including those with concentrated HIV epidemics. Key explanations could include the generally late diagnosis of HIV and presentation with advanced immune deterioration [18,19], suboptimal adherence to ART and retention in care, and risk of development of TB even among PLHIV receiving effective ART [20].

The lack of difference in hospitalizations because of TB before and after 2009 highlights the continuous burden of TB on HIV-associated care and points to the need for earlier ART initiation and expanded ART and IPT coverage. The results from the recent START trial [21] add to the results from the HPTN 052 study [22] in showing that early initiation of ART has a major impact on TB incidence. Further, recent results from the TEMPRANO study [23] showed that IPT has an additive efficacy with respect to the prevention of TB. These results add to other data that suggest that the two therapies should be given concomitantly [24–26] In this review, no studies reported IPT use at time of admission, and while this may reflect a limitation in reporting it is also consistent with the other studies showing that the scale of implementation of preventive therapy for TB among PLHIV is still limited [27].

We found very high rates of in-hospital mortality because of TB. Although recommendations are in place to start ART in all individuals with a TB diagnosis [28], only 38% of patients hospitalized with TB were on ART at admission and this may have contributed to the high in-hospital mortality.

The burden of TB-associated hospital admission could be reduced through earlier diagnosis of TB in the outpatient setting [29]. There are challenges in the diagnosis of TB among PLHIV [30], which can be improved by using Xpert MTB/RIF as the first line test in those suspected of TB [31]. However, the scale-up and uptake of Xpert MTB/RIF has so far been limited [32] despite its capacity to substantially reduce the delay in diagnosis and treatment initiation [32,33]. Enhancing the symptom-based routine TB screening among PLHIV and expediting the diagnosis of TB using Xpert MTB/RIF are important priorities.

Strengths of this review include a broad search strategy across multiple databases that allowed a large number of studies across a range of settings to be identified. We used random effects models and undertook a number of analyses to explore the extent of heterogeneity by region, age, diagnostic approach and use of ART at admission. The main limitation of this review is the limited reporting by studies of ART and IPT status, TB diagnostic method used, TB drug resistance patterns and the occurrence of TB-immune reconstitution inflammatory syndrome, which could potentially explain observed differences in TB-associated morbidity and mortality between studies. Another important limitation is that few studies reported how cause of mortality was assessed. A recent review of autopsy studies found that TB was the cause of death in 37% (26 to 49%) of adult deaths related to HIV/AIDS [7], which is higher than the pooled proportion of TB-associated deaths in this study (25%, 19 to 31%); this difference may reflect both more reliable ascertainment of cause of death and the fact that a proportion of TB-associated deaths occur without prior hospitalization. Finally, the quality of the individual studies contributing to this review was variable, with over half (34 studies) being retrospective designs, and eight being conference abstracts.

In conclusion, this review underscores the need to strengthen efforts to improve early diagnosis of HIV, prompt linkage to care and provision of ART and strengthen TB prevention activities for people living with HIV, notably through intensified case finding, provision of IPT and infection control [34]. Considering the high mortality of HIV-positive hospitalized patients who are found to have TB, national HIV and TB programmes should embark on joint planning in order to ensure capacity to diagnose TB among adults and children living with HIV and to provide effective treatment when TB is diagnosed.

## Supplementary Material

TB as a cause of hospitalization and in-hospital mortality among people living with HIV worldwide: a systematic review and meta-analysis
