# Supplementary material for: TB as a cause of hospitalization and in-hospital mortality among people living with HIV worldwide: a systematic review and meta-analysis
Source: J Int AIDS Soc. 2016 Jan 12;19(1):20714. doi: 10.7448/IAS.19.1.20714 (PMC4712323; doi:10.7448/IAS.19.1.20714)
Supplement: TB as a cause of hospitalization and in-hospital mortality among people living with HIV worldwide: a systematic review and meta-analysis [file JIAS-19-20714-s001.doc]

**Supplementary Table 1: Characteristics of included studies**

| **Study** | **Setting** | **Study design** | **Country** | **WHO**  **Region** | **Period** | **Mid point** | **Age**  **Median/**  **mean*** | **Number of patients** | **Number hospitalized** | **Number who died** | **Diagnostic method** |
| --- | --- | --- | --- | --- | --- | --- | --- | --- | --- | --- | --- |
| **Adults** |  |  |  |  |  |  |  |  |  |  |  |
| Adlakha^1^ | University hospital ICU, London | Retrospective cohort | UK | EURO | 2006-2009 | 2007 | 41 | 90 | 109 | 20 | NR |
| Agaba^2^ | Tertiary teaching hospital, Jos | Retrospective cohort | Nigeria | AFRO | 2007 | 2007 | 35* | 354 | 354 | 123 | Clinical + At Least 1 Laboratory Finding |
| Akinkuotu^3^ | Central Hospital, Llongwe | Prospective cohort | Malawi | AFRO | 2008-9 | 2007 | 36.5* | 537 | 537 | 130 | Clinical + At Least 1 Laboratory Finding |
| Apetse^4^ | 22 hospitals nationwide | Prospective cohort | Togo | AFRO | 2008 | 2008 | 37* | 714 | 714 | 163 | Clinical + At Least 1 Laboratory Finding |
| Balkhair^5^ | University hospital, Muscat | Retrospective cohort | Oman | EMRO | 1999-2008 | 2004 | 37.5* | 77 | 77 | 10 | Clinical + At Least 1 Laboratory Finding |
| Banda^6^ | University hospital, Lusaka | Retrospective cohort | Zambia | AFRO | 2010 | 2010 | 35.6 | 142 | 142 | NS | NR |
| Barbier^7^ | 40 ICUs, Paris | Administrative database | France | EURO | 2008-2010 | 2009 | 43 | 1348 | 1348 | 1707 | NR |
| Bolokadze^8^ | Infectious disease inpatient clinic, Tbilisi | Prospective cohort | Georgia | EURO | 2006-8 | 2007 | 36 | 388 | 388 | NS | Clinical + At Least 1 Laboratory Finding |
| Pecanha de Castro^9^ | Central hospital, Salvador | Retrospective cohort | Brazil | AMRO | 2011-2012 | 2011 | 21-40 | 50 | 50 | NS | NR |
| Chakraborty^10^ | University hospital, Kolkata | Retrospective cohort | India | SEARO | 2006-7 | 2006 | 35 | 125 | 125 | 7 | Sputum Smear |
| Cordova^11^ | University hospital, Montevideo | Retrospective cohort | Uruguay | AMRO | 2004-2007 | 2006 | 37.5 | 116 | 116 | 11 | Clinical + At Least 1 Laboratory Finding |
| Dai^12^ | Referral hospital, Beijing | Retrospective cohort | China | WPRO | 2009-11 | 2010 | 36 | 60 | 60 | 8 | Clinical + At Least 1 Laboratory Finding |
| Dias^13^ | 43 public hospitals | Administrative data# | Portugal | EURO | 2005-2007 | 2006 | 41.6 | 12078 | 12078 | 1883 | NR |
| Fortes Déguénonvo^14^ | Central hospital, Dakar | Retrospective cohort | Senegal | AFRO | 2007-8 | 2008 | 41 | 527 | 527 | 231 | NR |
| Guerro^15^ | General hospital, southern Brazil | Cross-sectional study | Brazil | AMRO | 2007-12 | 2009 | 40.9* | 230 | 550 | 103 | NR |
| Hajiabdolbaghi^16^ | General hospital, Tehran | Cross sectional study | Iran | EMRO | 2009-12 | 2011 | 36.6* | 555 | 555 | 101 | NR |
| Japiassu^17^ | ICU, Rio de Janeiro | Prospective cohort | Brazil | AMRO | 2006-8 | 2007 | 40* | 88 | 88 | 43 | NR |
| Kang^18^ | Multisite | Prospective cohort | Korea | SEARO | 2006-2013 | 2010 | 46 | 1096 | 1096 | 26 | NR |
| Kifle^19^ | University hospital, Gondor | Cross sectional study | Ethiopia | AFRO | NS |  | 25-34 | 360 | 360 | NS | Clinical + At Least 1 Laboratory Finding |
| Kra^20^ | University hospital, Treichville | Retrospective cohort | Ivory Coast | AFRO | 2006-7 | 2007 | 39* | 447 | 447 | 109 | NR |
| Lewden^21^ | 6 teaching hospitals | Multisite prospective cohort | Benin, Burkina Faso, Ivory Coast, Mali, Senegal | AFRO | 2010 | 2010 | 40 | 823 | 823 | 315 | Clinical + At Least 1 Laboratory Finding |
| Lucero^22^ | University hospital, Barcelona | Prospective cohort | Spain | EURO | 1996-2007 | 2004 | 43 | 182 | 336 | 8 | NR |
| Luma^23^ | General hospital, Douala | Retrospective cohort | Cameroon | AFRO | 2004-9 | 2007 | 38.1* | 672 | 672 | 147 | Clinical |
| Luz^24^ | 9 public hospitals, France & 1 clinical research institute, Rio de Janeiro | Prospective cohort | Brazil & France | AMRO and EURO | 2000-8 | 2005 | >18 years | 2238 | 4689 | 891 | NR |
| Matin^25^ | Referral hospital, Dhaka | Retrospective cohort | Bangladesh | SEARO | 2008-10 | 2009 | 35 | 109 | 109 | 13 | Clinical |
| Medrano^26^ | Multiple hospital ICUs | Administrative data# | Spain | EURO | 2005-10 | 2008 | 43 | 1891 | 1891 | 1531 | Clinical + At Least 1 Laboratory Finding |
| Melo-Uribe^27^ | 1 hospital | Autopsy study | Colombia | AMRO | 2004-2013 | 2009 | 36.6* | 155 | 155 | 155 | Sputum smear |
| Metallidis^28^ | University hospital, Thessaloniki | Retrospective cohort | Greece | EURO | 1998-2008 | 2003 | 33* | 558 | 558^$^ | 43 | Sputum smear + culture |
| Meyer Rath^29^ | 1 urban (Soweto) and 1 rural (Mpumalanga) hospital | Prospective cohort | South Africa | AFRO | 2003-10 | 2007 | 33 | 534 | 534 | NS | NR |
| Miranda^30^ | Referral hospital, Lisbon | Retrospective cohort | Portugal | EURO | 2009-2011 | 2010 | 46* | 617 | 617 | 61 | NR |
| Moazen^31^ | Hospital, Zahedan | Retrospective cohort | Iran | EMRO | 2000-2010 | 2005 | NS | 65 | 65 | NS | NR |
| Morquin^32^ | University hospital ICU | Retrospective cohort | France | EURO | 1997-2008 | 2003 | 43* | 98 | 103 | 47 | NR |
| Namutebi^33^ | Referral hospital, Kampala | Prospective cohort | Uganda | AFRO | 2011 | 2011 | 34 | 201 | 201 | 42 | Clinical + treatment response |
| Neves^34^ | University hospital, Sao Paulo | Retrospective cohort | Brazil | AMRO | 2007-2008 | 2008 | 39* | 41 | 41 | NS | NR |
| Ogoina^35^ | Tertiary hospital, northern Nigeria | Retrospective cohort | Nigeria | AFRO | 2006-9 | 2008 | 36 | 207 | 207 | 67 | Clinical + At Least 1 Laboratory Finding |
| Ondounda^36^ | Military hospital, Libreville | Retrospective cohort | Gabon | AFRO | 2008-10 | 2009 | 40 | 289 | 289 | 80 | NR |
| Paudel^37^ | Hospital, Kailali | Cross-sectional study | Nepal | SEARO | 2009 | 2009 | 31-40 | 66 | 66 | 3 | Clinical + At Least 1 Laboratory Finding |
| Ribeiro^38^ | Referral hospital, Rio de Janeiro | Prospective cohort | Brazil | AMRO | 2000-10 | 2007 | 41 | 1094 | 2443 | 249 | NR |
| Rodger^39^ | 115 clinical sites | Web-based questionnaire | UK | EURO | 2007 | 2007 | 35* | 255 | 255 | NS | NR |
| Saldarriaga-Arenas^40^ | Hospital, Pereira | Prospective cohort | Colombia | AMRO | 2010-2011 | 2011 | 35.5* | 76 | 76 | 14 | Clinical + At Least 1 Laboratory Finding |
| Seng^41^ | 10 hospitals, Paris | Prospective cohort# | France | EURO | 2011 | 2011 | 48 | 1489 | 3013 | NS | NR |
| Shahani^42^ | 2 hospitals, Texas | Prospective cohort# | USA | AMRO | 2006-7 | 2006 | 65% aged 30-49 | 65 | 65 | NS | NR |
| Shrosbree^43^ | University hospital ICU, London | Retrospective cohort | UK | EURO | 2000-9 | 2005 | 38* | 118 | 122 | 45 | NR |
| Singh^44^ | Referral hospital, Bathinda | Prospective cohort | India | SEARO | 2006-9 | 2008 | 41* | 416 | 416 | NS | NR |
| Takalkar^45^ | Community care centre, Narketpally | Retrospective cohort | India | SEARO | 2008-2009 | 2008 | 26-45 | 110 | 110 | NS | Sputum smear |
| Thinyane^46^ | Tertiary hospital, Maseru | Prospective cohort | Lesotho | AFRO | 2010 | 2010 | 34 | 105 | 105 | 32 | NR |
| Tittle^47^ | 1 tertiary hospital (London) and 4 infectious disease wards (Italy) | Retrospective cohort# | UK & Italy | EURO | 2012 | 2012 | 49 | 731 | 731 | 30 | NR |
| Traore^48^ | University hospital, Bamako | Prospective cohort | Mali | AFRO | 2009-10 | 2009 | 37.8* | 352 | 352 | 152 | NR |
| Wen^49^ | University hospital, Shenyang | Prospective cohort | China | WPRO | 2010-12 | 2011 | 41* | 141 | 141 | 30 | Clinical + At Least 1 Laboratory Finding |
| Whitehorn^50^ | University hospital, London | Retrospective cohort | UK | EURO | 2007-9 | 2008 | 40 | 29 | 29 | 7 | NR |
| Xiao 2013^51^ | Tertiary care, Beijing | Retrospective cohort | China | WPRO | 2009-12 | 2010 | 39* | 834 | 834 | 142 | Clinical + At Least 1 Laboratory Finding |
| **Children** |  |  |  |  |  |  |  |  |  |  |  |
| Bwakura-Dangarembiz^52^ | 3 hospitals in Uganda and 1 in Zimbabwe | RCT | Uganda, Zimbabwe | AFRO | 2009-12 | 2010 | 7.5 | 168 | 168 | 5 | Sputum smear + culture |
| Collins^53^ | 40 public hospitals | Prospective cohort | Thailand | SEARO | 1999-2009 | 2004 | 7 | 211 | 451 | 23 | NR |
| Desmonde^54^ | Public hospital, Abidjan | Retrospective cohort | Ivory Coast | AFRO | 2004-9 | 2007 | 4.5 | 38 | 38 | NS | Clinical + At Least 1 Laboratory Finding |
| Dicko^55^ | 5 paediatric hospitals | Retrospective cohort | Mali, Benin, Senegal, Burkina Faso, Ghana | AFRO | 2010 | 2010 | 3 | 155 | 155 | 25 | Clinical |
| Dramowski^56^ | Public hospital, Soweto | Retrospective cohort | South Africa | AFRO | 2007 | 2007 | 1 | 440 | 440 | 53 | Clinical |
| Ferrand^57^ | 2 public hospitals, Harare | Prospective cohort | Zimbabwe | AFRO | 2007-8 | 2007 | 13 | 139 | 139 | 32 | Sputum smear + culture |
| Hattasingh^58^ | Public hospital, Khon Kaen province | Retrospective cohort | Thailand | SEARO | 2007-2009 | 2008 | NR | 238 | 238 | NS | NR |
| Kwara^59^ | Teaching hospital, Korle-Bu | Retrospective cohort | Ghana | AFRO | 2007-8 | 2008 | 4.7* | 76 | 102 | 12 | Clinical + At Least 1 Laboratory Finding |
| Meyers^60^ | Public hospital, Soweto | Prospective cohort | South Africa | AFRO | 2010-11 | 2010 | 1.5 | 73 | 73 | 62 | Clinical |
| Moreira^61^ | Public children’s hospital Vitória-Espírito Santo | Retrospective cohort | Brazil | AMRO | 2001-11 | 2006 | 5 | 177 | 177 | 26 | Clinical |
| Nyandiko^62^ | University hospital, Eldoret | Retrospective cohort | Kenya | AFRO | 2002-8 | 2005 |  | 91 | 91 | 24 | Clinical |
| Shahrin^63^ | Specialized hospital, Dhaka | Prospective cohort | Bangladesh | SEARO | 2009-12 | 2010 | 7 | 24 | 24 | 1 | NR |
| Sudjaritruk^64^ | University hospital, Chiang Mai | Prospective cohort | Thailand | SEARO | 2003-9 | 2006 | 7* | 140 | 140 | 16 | Clinical |
| Viani^65^ | Tijuana General Hospital | Retrospective cohort | Mexico | AMRO | 1998-2007 | 2003 | 2* | 73 | 73 | 23 | Clinical + At Least 1 Laboratory Finding |
| Villalobos-Acosta^66^ | 1 hospital | Retrospective cohort | Mexico | AMRO | 1989-2008 | 1999 | 3-5 | 483 | 483 | 22 | NR |

#Discharge diagnosis; ^$^225 patients <50 years and 28 patients >50 years presenting with a major event were not hospitalized; ICU, intensive care unit; NR, not reported; NS, not stated; RCT, randomized controlled trial

**References**

1. Adlakha A, Pavlou M, Walker DA, et al. Survival of HIV-infected patients admitted to the intensive care unit in the era of highly active antiretroviral therapy. *International Journal of STD & AIDS* 2011; **22**(9): 498-504.

2. Agaba PA, Digin E, Makai R, et al. Clinical characteristics and predictors of mortality in hospitalized HIV-infected Nigerians. *Journal of Infection in Developing Countries* 2011; **5**(5): 377-82.

3. Akinkuotu A, Roemer E, Richardson A, et al. In-hospital mortality rates and HIV: a medical ward review, Lilongwe, Malawi. *International Journal of STD & AIDS* 2011; **22**(8): 465-70.

4. Apetse K, Assogba K, Kevi K, Balogou AA, Pitche P, Grunitzky E. [Opportunistic infections of the HIV/AIDS in adults in hospital settings in Togo]. *Bulletin de la Societe de Pathologie Exotique* 2011; **104**(5): 352-4.

5. Balkhair AA, Al-Muharrmi ZK, Ganguly S, Al-Jabri AA. Spectrum of AIDS Defining Opportunistic Infections in a Series of 77 Hospitalised HIV-infected Omani Patients. *Sultan Qaboos University Medical Journal* 2012; **12**(4): 442-8.

6. Banda J, Mweemba A, Siziya S, Mweene M, Andrews B, Lakhi S. Prevalence and Factors Associated with Renal Dysfunction in HIV Positive and Negative Adults at the University Teaching Hospital, in Lusaka. *Medical Journal of Zambia* 2010; **37**(3): 136-42.

7. Barbier F, Roux A, Canet E, et al. Temporal trends in critical events complicating HIV infection: 1999-2010 multicentre cohort study in France. *Intensive Care Medicine* 2014; **40**(12): 1906-15.

8. Bolokadze N, Gabunia P, Ezugbaia M, Gatserelia L, Khechiashvili G. Neurological complications in patients with HIV/AIDS. *Georgian medical news* 2008; (165): 34-8.

9. Pecanha de Castro A, Magalhaesa M, Lirio M, Paste A. Perfil Socioeconomico e clinico dos pacientes internados com HIV/AIDS em hospital de Salvador, Bahio. *Revista Baiana de Saúde Pública* 2013. 37; S1: 22-132.

10. Chakraborty N, Mukherjee A, Santra S, et al. Current trends of opportunistic infections among HIV-seropositive patients from Eastern India. *Japanese Journal of Infectious Diseases* 2008; **61**(1): 49-53.

11. Cordova V, Lopez M, Arteta Z, Correa F. VIH-SIDA en la clínica médica: descripción de una población hospitalaria. *Arch Med interna* 2009; XXXI;4:99-101.

12. Dai L, Mahajan SD, Guo C, et al. Spectrum of central nervous system disorders in hospitalized HIV/AIDS patients (2009-2011) at a major HIV/AIDS referral center in Beijing, China. *Journal of the Neurological Sciences* 2014; **342**(1-2): 88-92.

13. Dias SS, Andreozzi V, Martins MO, Torgal J. Predictors of mortality in HIV-associated hospitalizations in Portugal: a hierarchical survival model. *BMC Health Services Research* 2009; **9**: 125.

14. Fortes Deguenonvo L, Manga NM, Diop SA, et al. [Current profile of HIV-infected patients hospitalized in Dakar (Senegal)]. *Bulletin de la Societe de Pathologie Exotique* 2011; **104**(5): 366-70.

15. Guerro AC, Andretta IB, Bello SL, Trevisol DJ, Schuelter-Trevisol F. Causes of hospital admission of AIDS patients in southern Brazil, 2007 to 2012. *Revista da Sociedade Brasileira de Medicina Tropical* 2014; **47**(5): 632-6.

16. Hajiabdolbaghi M, Jafari S, Mansouri S, Hedayat Yaghoobi M. Hospitalizations and its related factors in HIV/AIDS patients in Tehran, Iran. *Medical journal of the Islamic Republic of Iran* 2014; **28**: 70.

17. Japiassu AM, Amancio RT, Mesquita EC, et al. Sepsis is a major determinant of outcome in critically ill HIV/AIDS patients. *Critical Care* 2010; **14**(4): R152.

18. Kang MW, Kim YJ, Kim SI, Korean K. Opportunistic diseases among HIV-infected patients: Korea HIV/AIDS Cohort study, 2006-2013. International Journal of Infectious Diseases. Conference: 16th International Congress on Infectious Diseases, 2014.

19. Kifle D. Prevalence and immunological correlates of opportunistic infections among HIV patients attending at art clinic of university of gondar hospital. Sexually Transmitted Infections. Conference: STI and AIDS World Congress 2013 Vienna Australia.

20. Kra O, Aba YT, Yao KH, et al. [Clinical, biological, therapeutic and evolving profile of patients with HIV infection hospitalized at Infectious and tropical diseases unit in Abidjan (Ivory Coast)]. *Bulletin de la Societe de Pathologie Exotique* 2013; **106**(1): 37-42.

21. Lewden C, Drabo YJ, Zannou DM, et al. Disease patterns and causes of death of hospitalized HIV-positive adults in West Africa: a multicountry survey in the antiretroviral treatment era. *Journal of the International AIDS Society* 2014; **17**: 18797.

22. Lucero C, Torres B, Leon A, et al. Rate and predictors of non-AIDS events in a cohort of HIV-infected patients with a CD4 T cell count above 500 cells/mm(3). *AIDS Research and Human Retroviruses* 2013; **29**(8): 1161-7.

23. Luma HN, Tchaleu BC, Temfack E, et al. HIV-Associated Central Nervous System Disease in Patients Admitted at the Douala General Hospital between 2004 and 2009: A Retrospective Study. *AIDS Research and Treatment* 2013; **2013**: 709810.

24. Luz PM, Bruyand M, Ribeiro S, et al. AIDS and non-AIDS severe morbidity associated with hospitalizations among HIV-infected patients in two regions with universal access to care and antiretroviral therapy, France and Brazil, 2000-2008: hospital-based cohort studies. *BMC Infectious Diseases* 2014; **14**: 278.

25. Matin N, Shahrin L, Pervez MM, et al. Clinical profile of HIV/AIDS-infected patients admitted to a new specialist unit in Dhaka, Bangladesh--a low-prevalence country for HIV. *Journal of Health, Population, and Nutrition* 2011; **29**(1): 14-9.

26. Medrano J, Alvaro-Meca A, Boyer A, Jimenez-Sousa MA, Resino S. Mortality of patients infected with HIV in the intensive care unit (2005-2010): significant role of chronic hepatitis C and severe sepsis. *Critical Care* 2014; **18**(4): 475.

27. Melo-Uribe MA, Mantilla-Hernandez JC, Idarraga JD. HIV/AIDS-related opportunist infections, base on analysis of autopsy cases. 103rd Annual Meeting of the United States and Canadian Academy of Pathology, USCAP 2014 San Diego.

28. Metallidis S, Tsachouridou O, Skoura L, et al. Older HIV-infected patients--an underestimated population in northern Greece: epidemiology, risk of disease progression and death. *International Journal of Infectious Diseases* 2013; **17**(10): e883-91.

29. Meyer-Rath G, Brennan AT, Fox MP, et al. Rates and cost of hospitalization before and after initiation of antiretroviral therapy in urban and rural settings in South Africa. *Journal of Acquired Immune Deficiency Syndromes* 2013; **62**(3): 322-8.

30. Miranda A, Fernandes D, Peres S, et al. Hospital admissions of HIV-infected patients at a Lisbon reference centre: Comparison among previously known and in-ward HIV-diagnosed patients. Journal of the International AIDS Society. Conference: 11th International Congress on Drug Therapy in HIV Infection Glasgow United Kingdom. 2012.

31. Moazen J, Sharifi-Mood B, Metanat M. Spectrum of opportunistic infectious diseases among hospitalized patients with HIV/AIDS in Boo-Ali Hospital, Zahedan-Southeastern Iran. Tropical Medicine and International Health. Conference: 7th European Congress on Tropical Medicine and International Health Barcelona Spain. 2011.

32. Morquin D, Le Moing V, Mura T, et al. Short- and long-term outcomes of HIV-infected patients admitted to the intensive care unit: impact of antiretroviral therapy and immunovirological status. *Annals of Intensive Care* 2012; **2**(1): 25.

33. Namutebi AM, Kamya MR, Byakika-Kibwika P. Causes and outcome of hospitalization among HIV-infected adults receiving antiretroviral therapy in Mulago hospital, Uganda. *African Health Sciences* 2013; **13**(4): 977-85.

34. Neves FF, Figueiredo JF, Jordao Junior AA, Vannucchi H. [Influence of acute-phase inflammatory response on serum levels of retinol and retinol binding protein in HIV/AIDS patients]. *Revista da Sociedade Brasileira de Medicina Tropical* 2010; **43**(1): 23-6.

35. Ogoina D, Obiako RO, Muktar HM, et al. Morbidity and Mortality Patterns of Hospitalised Adult HIV/AIDS Patients in the Era of Highly Active Antiretroviral Therapy: A 4-year Retrospective Review from Zaria, Northern Nigeria. *AIDS Research and Treatment* 2012; **2012**: 940580.

36. Ondounda M, Magne C, Mounguengui D, Gaudong Mbethe L, Nzenze JR. [Morbidity and mortality in HIV-infected patients in the Military Hospital in Libreville (Gabon)]. *Medecine et Sante Tropicales* 2012; **22**(3): 334-5.

37. Paudel BN, Dhungana GP. Scenario of HIV/AIDS patients in a government hospital of Nepal. *Journal of Nepal Health Research Council* 2010; **8**(2): 103-6.

38. Ribeiro SR, Luz PM, Campos DP, et al. Incidence and determinants of severe morbidity among HIV-infected patients from Rio de Janeiro, Brazil, 2000-2010. *Antiviral Therapy* 2014; **19**(4): 387-97.

39. Rodger AJ, Curtis H, Sabin C, et al. Assessment of hospitalizations among HIV patients in the UK: a national cross-sectional survey. *International Journal of STD & AIDS* 2010; **21**(11): 752-4.

40. Saldarriaga-Arenas PA, Rodriguez-Morales AJ. Epidemiology of opportunistic diseases in AIDS patients from Pereira municipality, Colombia, 2010-2011. *Journal of Infection and Public Health* 2013; **6**(6): 496-8.

41. Seng R, Mutuon P, Riou J, et al. Changing causes of hospitalizations of people living with HIV, within 10 hospitals in the southern Paris area (“COREVIH Ile de France Sud”). 19th International Workshop on HIV Observational Databases. Catania, Sicily, 26-28 March 2015.

42. Shahani L, Hartman C, Troisi C, Kapadia A, Giordano TP. Causes of hospitalization and perceived access to care among persons newly diagnosed with HIV infection: implications for HIV testing programs. *AIDS Patient Care and STDs* 2012; **26**(2): 81-6.

43. Shrosbree J, Campbell LJ, Ibrahim F, et al. Late HIV diagnosis is a major risk factor for intensive care unit admission in HIV-positive patients: a single centre observational cohort study. *BMC Infectious Diseases* 2013; **13**: 23.

44. Singh R, Kaur M, Arora D. Neurological complications in late-stage hospitalized patients with HIV disease. *Annals of Indian Academy of Neurology* 2011; **14**(3): 172-7.

45. Takalkar AA, Saiprasad GS, Prasad VG, Madhekar NS. Study of Opportunistic Infections In HIV Seropositive Patients Admitted to Community Care centre (CCC), KIMS Narketpally. *Biomedical Research* 2012. 23: 139-142.

46. Thinyane K, Cooper V. Clinical Profiles of HIV-Infected, HAART-Naive Patients Admitted to a Tertiary Level Hospital in Maseru, Lesotho. *The Internet Journal of Infectious Diseases*; 11: 1.

47. Tittle V, Cenderello G, Pasa A, et al. A comparison of inpatient admissions in 2012 from two European countries. *Journal of the International AIDS Society* 2014; **17**(4 Suppl 3): 19712.

48. Traore AM, Minta DK, Fomba M, et al. [Epidemiological, clinical and evolving HIV-positive patients referred to the University Hospital of Point G, Bamako, Mali.]. *Bulletin de la Societe de Pathologie Exotique* 2013.

49. Wen Y, Zhou Y, Wang W, et al. Baseline factors associated with mortality within six months after admission among hospitalized HIV-1 patients in Shenyang, China. *Internal medicine* 2014; **53**(21): 2455-61.

50. Whitehorn J, Edwards SG, Cartledge JD, Miller RF. Outcome of HIV-infected patients transferred to a specialist inpatient unit. *International journal of STD & AIDS* 2011; **22**(4): 225-7.

51. Xiao J, Gao G, Li Y, et al. Spectrums of opportunistic infections and malignancies in HIV-infected patients in tertiary care hospital, China. *PloS one* 2013; **8**(10): e75915.

52. Bwakura-Dangarembizi M, Kendall L, Bakeera-Kitaka S, et al. A randomized trial of prolonged co-trimoxazole in HIV-infected children in Africa. *The New England Journal of Medicine* 2014; **370**(1): 41-53.

53. Collins IJ, Cairns J, Jourdain G, et al. Hospitalization trends, costs, and risk factors in HIV-infected children on antiretroviral therapy. *Aids* 2012; **26**(15): 1943-52.

54. Desmonde S, Coffie PA, Aka EA, et al. Health care resource utilization in untreated HIV-infected children in a pediatric programme, Abidjan, Cote d'Ivoire, 2004-2009. *Journal of Acquired Immune Deficiency Syndromes* 2013; **62**(1): e14-21.

55. Dicko F, Desmonde S, Koumakpai S, et al. Reasons for hospitalization in HIV-infected children in West Africa. *Journal of the International AIDS Society* 2014; **17**: 18818.

56. Dramowski A, Coovadia A, Meyers T, Goga A. Identifying missed opportunities for early intervention among HIV-infected paediatric admissions at Chris Hani Baragwanath Hospital, Soweto, South Africa. Southern Africa Journal of HIV Medicine, December 2011. 16-23.

57. Ferrand RA, Bandason T, Musvaire P, et al. Causes of acute hospitalization in adolescence: burden and spectrum of HIV-related morbidity in a country with an early-onset and severe HIV epidemic: a prospective survey. *PLoS Medicine* 2010; **7**(2): e1000178.

58. Hattasingh W, Younis BM, Kosalaraksa P, Limkittikul K. Characteristics of opportunistic infections in HIV-infected children during pre-HAART and HAART era in Srinagarind hospital, Thailand. International Journal of Infectious Diseases. Conference: 15th International Congress on Infectious Diseases, ICID 2012 Bangkok Thailand. 2012.

59. Kwara A, Shah D, Renner LA. Outcome of hospital admissions in HIV-infected children at the Korle Bu Teaching Hospital, Accra, Ghana. *West African Journal of Medicine* 2010; **29**(6): 379-83.

60. Meyers T, Dramowski A, Schneider H, Gardiner N, Kuhn L, Moore D. Changes in pediatric HIV-related hospital admissions and mortality in Soweto, South Africa, 1996-2011: light at the end of the tunnel? *Journal of Acquired Immune Deficiency Syndromes* 2012; **60**(5): 503-10.

61. Moreira-Silva SF, Zandonade E, Frauches DO, et al. Comorbidities in children and adolescents with AIDS acquired by HIV vertical transmission in Vitoria, Brazil. *PloS ONE* 2013; **8**(12): e82027.

62. Nyandiko WM, Mwangi A, Ayaya SO, et al. Characteristics of HIV-infected children seen in Western Kenya. *East African Medical Journal* 2009; **86**(8): 364-73.

63. Shahrin L, Leung DT, Matin N, Kawser CA, Pervez MM, Chisti MJ. Clinical profile of hospitalized HIV-infected children in Bangladesh, a low-HIV-prevalence country. *Paediatrics and International Child Health* 2014; **34**(2): 133-7.

64. T Sudjaritruk, P Oberdorfer, T Puthanakit, T Sirisanthana and V Sirisanthana. Causes of first hospitalization among 1121 HIV-infected children: comparison of the pre-*Pneumocystis jiroveci* pneumonia prophylaxis, pre-antiretroviral therapy and antiretroviral therapy periods. *Int J STD AIDS* 2012 23: 335.

65. Viani RM, Araneta MR, Lopez G, Chacon-Cruz E, Spector SA. Clinical Outcomes and Hospitalizations among Children Perinatally Infected with HIV-1 in Baja California, Mexico. *Journal of the International Association of Physicians in AIDS Care* 2011; **10**(4): 223-8.

66. Villalobos-Acosta C, Plascencia-Gómez E, Romano-Mazzotti L, Pavía-Ruz N. Trends and survival in HIV-infected children and adolescents management with highly active antiretroviral therapy. *Bol Med Hosp Infant Mex* 2009. 66: 314-24.

**Supplementary Table 2: Search strategy**

| 1 | (Hospitalization [mesh] OR hospitaliz*[tiab] OR hospitalis*[tiab] OR rehospitalis* [tiab] OR rehospitaliz*[tiab] OR admit*[tiab] OR admis*[tiab]) |
| --- | --- |
| 2 | HIV Infections[MeSH] OR HIV[MeSH] OR hiv[tw] OR hiv-1*[tw] OR hiv-2*[tw] OR hiv1[tw] OR hiv2[tw] OR hiv infect*[tw] OR human immunodeficiency virus[tw] OR human immunedeficiency virus[tw] OR human immuno-deficiency virus[tw] OR human immune-deficiency virus[tw] OR ((human immun*) AND (deficiency virus[tw])) OR acquired immunodeficiency syndrome[tw] OR acquired immunedeficiency syndrome[tw] OR acquired immuno-deficiency syndrome[tw] OR acquired immune-deficiency syndrome[tw] OR ((acquired immun*) AND (deficiency syndrome[tw])) OR "sexually transmitted diseases, viral" [MESH:NoExp] |
| 4 | #1 AND #2 |
